# CDK4 Amplification in Esophageal Squamous Cell Carcinoma Associated With Better Patient Outcome

**DOI:** 10.3389/fgene.2021.616110

**Published:** 2021-04-29

**Authors:** Jie Huang, Xiang Wang, Xue Zhang, Weijie Chen, Lijuan Luan, Qi Song, Hao Wang, Jia Liu, Lei Xu, Yifan Xu, Licheng Shen, Lijie Tan, Dongxian Jiang, Jieakesu Su, Yingyong Hou

**Affiliations:** ^1^Department of Pathology, Zhongshan Hospital, Fudan University, Shanghai, China; ^2^Department of Thoracic Surgery, Zhongshan Hospital, Fudan University, Shanghai, China; ^3^Department of Pathology, Zhongshan Hospital, School of Basic Medical Sciences, Fudan University, Shanghai, China

**Keywords:** esophageal squamous cell carcinoma, *CDK4* amplification, clinical stage, prognostic value, fluorescence *in situ* hybridization

## Abstract

In the present study, we aimed to investigate the clinical and prognostic values of *CDK4* amplification and improve the risk stratification in patients with esophageal squamous cell carcinoma. *CDK4* amplification was analyzed by fluorescence *in situ* hybridization using tissue microarray consisting of representative tissues of 520 patients with esophageal squamous cell carcinoma, and its correlation with clinicopathological features and clinical outcomes were evaluated. *CDK4* amplification was found in 8.5% (44/520) of patients with esophageal squamous cell carcinoma. *CDK4* amplification was negatively correlated with disease progression (*P* = 0.003) and death (*P* = 0.006). Patients with *CDK4* amplification showed a significantly better disease-free survival (*P* = 0.016) and overall survival (*P* = 0.023) compared with those patients without *CDK4* amplification. When patients were further stratified into I–II stage groups and III–IV stage groups, *CDK4* amplification was significantly associated with both better disease-free survival (*P* = 0.023) and overall survival (*P* = 0.025) in the I–II stage group rather than the III–IV stage group. On univariate and multivariate analysis, invasive depth and *CDK4* amplification were associated with disease-free survival and overall survival. Taken together, *CDK4* amplification was identified as an independent prognostic factor for survival, which could be incorporated into the tumor–node–metastasis staging system to refine risk stratification of patients with esophageal squamous cell carcinoma.

## Introduction

Esophageal cancer (EC) is a lethal digestive tract malignancy with a poor prognosis, and an increasing incidence and mortality rate worldwide (Malhotra et al., 2017). There are two main histological types of EC: esophageal squamous cell cancer (ESCC) and esophageal adenocarcinoma (EAC), which have significant differences in pathogenesis, epidemiology, and risk factors (Rustgi and El-Serag, 2014; Arnold et al., 2015). ESCC usually occurs in flat cells lining the upper two thirds of the esophagus, predominantly in Africa and eastern Asia (especially in China), and smoking is the main risk factor (Lin et al., 2013; Rustgi and El-Serag, 2014), while EC mostly originates from the Barrett mucosa in the lower third of the esophagus and is prevalent in many developed countries (Edgren et al., 2013; Rustgi and El-Serag, 2014; Arnold et al., 2016). In addition to environmental and external factors, genetic factors may also contribute to the development of a specific type of EC. Recently, whole-genome sequencing and genome-wide association studies have been undertaken to identify EC-related genetic alterations (Gao et al., 2014; Lin et al., 2014; Cancer Genome Atlas Research Network, Analysis Working Group: Asan University, Bc Cancer Agency, Brigham and Women’s Hospital, Broad Institute, Brown University, et al., 2017).

Cell cycle dysregulation induced by abnormal genetic alterations (mutations, deletions, or amplifications) occur frequently in human malignancies (Hanahan and Weinberg, 2011; Gampenrieder et al., 2016). The deregulation of the cyclin D1–CDK4/6–Rb pathway, which will trigger loss of cell cycle control, is one of the hallmarks of carcinogenesis (Asghar et al., 2015). The cyclin-dependent kinase 4 (*CDK4*) gene located in the chromosomal region 12q14.1 might have oncogenic potential similar to other G1 regulatory genes (Haas et al., 1997). CDK4 is initially identified as a catalytic subunit present in the CDK/cyclin D complex in the G1 phase of the cell cycle (Matsushime et al., 1992). CDK4 coupled with cyclin D1 (CCND1) phosphorylates the retinoblastoma protein 1 (RB1), which leads to the release of the transcription factor EF2 and subsequently enables the cell cycle progress from G1 to S phase (Harbour et al., 1999). Alterations of these key components have been implicated in the pathogenesis of multiple tumor types (An et al., 1999). Overexpression of CDK4 could induce uncontrolled cell growth and eventually lead to tumorigenesis; moreover, amplification of the *CDK4* gene, have been found in various cancers (Lee et al., 2014).

The perturbed cell cycle regulation pathway in ESCC mainly exhibited genetic alterations in the G1/S transition control, including mutations or deletions of *TP53*, *RB1*, *CDKN2A*, *CHEK1*, and *CHEK2*, and amplifications of *CDK4*, *CCND1*, *CDK6*, and *MDM2* (Song et al., 2014). Alterations of these genes, such as inactivation of *RB1* and *CDKN2A* and amplification of *CCND1*, *CDK6*, and *MDM2* have been well documented in ESCC (Huang et al., 2007; Baba et al., 2014; Jiang et al., 2020). To date, the prognostic significance of *CDK4* amplification in ESCC has not been described before. In this article, we describe *CDK4* amplification in ESCC by fluorescence *in situ* hybridization (FISH) and meticulously investigated the clinical and prognostic values of *CDK4* amplification in patients with ESCC to improve the risk stratification.

## Materials and Methods

### Patients and Tissues

This study retrospectively enrolled 520 ESCC patients who had undergone surgical resection in the Department of Thorax Surgery, Zhongshan Hospital, Fudan University (Shanghai, China), between January 2007 and November 2010. Patients who received preoperative antitumor therapy, including neoadjuvant therapy, chemotherapy, and radiotherapy or died within 3 months were excluded from the current study. Ethical approval was granted by the Human Research Ethics Committee of Zhongshan Hospital, Fudan University. Signed informed consent for the acquisition and use of patient tissue specimens and clinical data was obtained from each patient.

All specimens were reassessed independently by two pathologists using hematoxylin and eosin (HE)-stained sections to determine the tumor grade, differentiation, invasion depth, lymph node metastasis, vessel and nerve involvement, and disease stage, according to the American Joint Committee on Cancer guidelines for tumor–node–metastasis (TNM) classification (eighth edition). Patients’ clinicopathological characteristics such as gender, age, smoking, tumor location, and clinical stage were collected from medical records. After surgery, patients were followed up with endoscopy and computed tomographic scan of the thorax and abdomen every 3 months for the first year, every 6 months for the second year, and every 6–12 months thereafter. Follow-up data of those patients who did not have themselves examined in our hospital were obtained by telephone.

### Tissue Microarray

Tissue microarrays (TMAs) containing tumor tissues of the 520 patients under study were constructed as previously described (Shi et al., 2013). Briefly, the representative areas of 2 mm wide and 6 mm long with rich tumor cells were selected by two experienced pathologists according to HE-stained slides. The corresponding regions on archived formalin-fixed, paraffin-embedded (FFPE) tissue blocks were extracted, vertically planted into the recipient TMA blocks and then aggregated on the instrument.

### Fluorescence *in situ* Hybridization and Assessment

Dual-color FISH assay was conducted on the TMA sections of 5 μm thickness using *CDK4*-specific probe (Spectrum orange) together with a centromere-specific probe (Spectrum green) for chromosome 12 (*CEP12*) (Empire Genomics, Buffalo, NY) for assessment of *CDK4* amplification according to established laboratory protocol, as previously described (Zhang et al., 2014). FISH copy number evaluation was performed by two experienced pathologists blinded to patients’ clinicopathologic characteristics under a fluorescence microscope (BX43; Olympus, Tokyo, Japan) equipped with a DAPI/green/orange triple band pass filter and a Microscope Digital Camera (DP73; Olympus). At least 100 tumor cell nuclei of each ESCC sample were analyzed by counting orange signals for *CDK4* and green signals for *CEP12* under an oil microscope with a magnification of 1,000 times. Overlapping tumor nuclei were excluded from evaluation to avoid false-positive scoring. Then the average number of *CDK4* and *CEP12* signals and the ratio of *CDK4/CEP12* were calculated for each case. Amplification of *CDK4* was defined as a *CDK4/CEP12* ratio ≥2.0 or an average copy number of *CDK4* signals/tumor cell nucleus ≥5.0 or percentage of tumor cells containing large clusters of *CDK4* signal ≥10%, respectively, based on previously reported modified scoring algorithms for *HER2* and *c-MYC* (Wolff et al., 2007; Huang et al., 2019).

### Statistical Analysis

All the statistical analyses were carried out using SPSS 20.0 (SPSS Inc., Chicago, IL, United States). All *P*-values were two sided, and differences were considered statistically significant values of *P* < 0.05. Disease-free survival (DFS) was defined as the interval between surgical resection and recurrence, metastasis, or death from any cause. Overall survival (OS) was defined as the interval from date of curative surgery until death or last follow-up date. Correlations between *CDK4* amplification and clinicopathologic variables were analyzed using the Fisher exact test or Pearson χ^2^ test. The Kaplan–Meier method with log-rank test was applied to calculate the cumulative survival proportion for OS and DFS by *CDK4* amplification level and to determine if there were any significant differences between the survival curves. The Cox proportional hazard regression model was used to carry out the univariate and multivariate regression analyses, and the hazard ratio (HR) and 95% confidence intervals (CI) were determined.

## Results

### Patient Characteristics

Detailed clinicopathological characteristics of the study cohort including 520 ESCC specimens obtained for this study are summarized in Table 1. The median age of this cohort was 61 years (range, 34–83 years), of which 81.7% were men and 38.7% were smokers. By anatomic site, 44.0% of tumors were in the middle esophagus, whereas 51.2% of the tumors were in the upper and lower esophagus with a median tumor size of 3 cm (range, 0.3–10 cm). The tumor differentiation was defined as grade I in 20 (3.8%) patients, II in 292 (56.2%) patients, and III in 208 (40.0%) patients. Vessel and nerve invasions were presented in 111 (21.3%) and 177 (34.0%) tumors, respectively. Meanwhile, lymph node metastasis was observed in 238 (45.8%) of the patients. The depth of invasion was also evaluated. 15 (2.9%) cases were confined to the mucosa, 38 (7.3%) were in the submucosa, 115 (22.1%) were in the muscular layer, and 352 (67.7%) were beyond the muscular layer. Among these patients with ESCC, clinical stage was classified as I to II and III to IVb in 290 (55.8%) and 230 (44.2%) cases, respectively, according to the American Joint Committee on Cancer Staging Manual (eighth edition).

**TABLE 1:** Correlation between *CDK4* amplification and clinicopathological features in full cohort of patients with ESCC.

Clinicopathologic feature	No.	*CDK4* amplification		
		
		No	Yes	*P-*value
Sex				0.987
Female	95	87	8	
Male	425	389	36	
Age (years)				0.588
<60	221	204	17	
≥60	299	272	27	
Grade				0.403
I + II	312	283	29	
III	208	193	15	
Invasive depth				0.791
I–II	168	153	15	
III	352	323	29	
Vessel invasion				0.592
No	409	373	36	
Yes	111	103	8	
Nerve invasion				0.511
No	343	312	31	
Yes	177	164	13	
Lymph node metastasis				0.556
No	282	260	22	
Yes	238	216	22	
Site				0.768
Up	25	22	3	
Middle	229	211	18	
Down	241	220	21	
Smoking				0.748
No	319	293	26	
Yes	201	183	18	
Clinical stage				0.625
I–II	290	267	23	
III–IVb	230	209	21	
Disease progression				**0.003**
No	242	212	30	
Yes	278	264	14	
Death				**0.006**
No	251	221	30	
Yes	269	255	14	

*Grade: I, well differentiated; II, moderately differentiated; III, poorly differentiated. Invasive depth: I, confined to submucosal layer; II, invasion of muscular layer; III, beyond the muscularis.*

*The features/variables that make significant contribution are in bold.*

### Association Between *CDK4* Amplification and Clinicopathological Features

**FIGURE 1:**
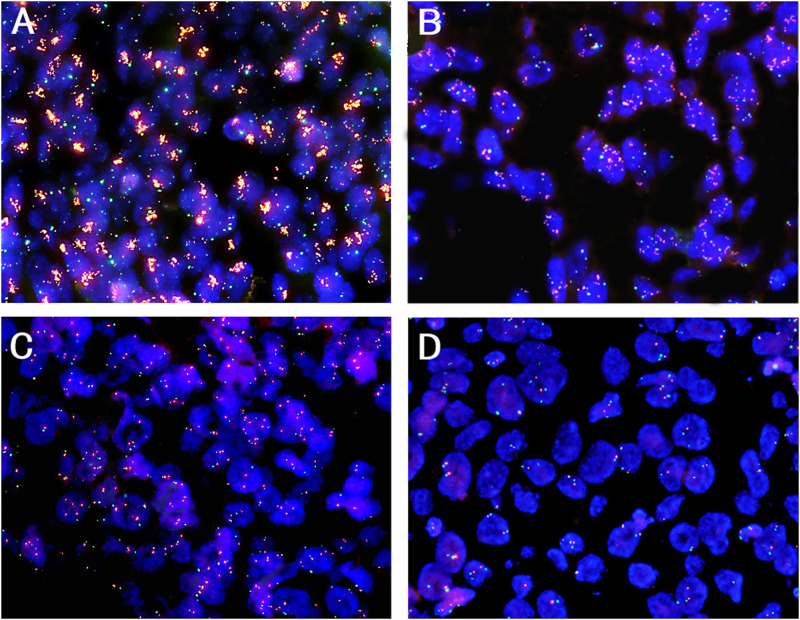
Representative patterns of *CDK4* gene (orange color) and CEP12 (green color) copy number status by FISH (original magnification ×1,000). **(A)** *CDK4* amplification, a *CDK4/CEP12* ratio ≥2.0; **(B)** *CDK4* amplification, an average copy number of *CDK4* signals/tumor cell nucleus ≥5.0; **(C)** *CDK4* non-amplification, low polysomy; **(D)** *CDK4* non-amplification, disomy.

All the patients were classified into two groups by using prespecified criteria for *CDK4* amplification based on previous studies (Wolff et al., 2007; Huang et al., 2019). *CDK4* amplification (a *CDK4/CEP12* ratio ≥2.0 or an average copy number of *CDK4* signals/tumor cell nucleus ≥5.0 or percentage of tumor cells containing large clusters of *CDK4* signal ≥10%) was found in 8.5% (44 of 520) of patients (Figures 1A,B), and other patients (91.5%, 476 of 520) showed non-amplification (low polysomy or disomy) (Figures 1C,D). The correlations between *CDK4* amplification and clinicopathological features are shown in Table 1. *CDK4* amplification status significantly correlated with disease progression (*P* = 0.003) and death (*P* = 0.006). There was no significant difference between *CDK4* amplification and *CDK4* non-amplification group regarding sex (*P* = 0.987), age (*P* = 0.588), grade (*P* = 0.403), invasive depth (*P* = 0.791), vessel (*P* = 0.592) and nerve invasions (*P* = 0.511), lymph node metastasis (*P* = 0.556), anatomic site (*P* = 0.768), smoking (*P* = 0.748), and clinical stage (*P* = 0.625).

### Survival Analyses

**FIGURE 2:**
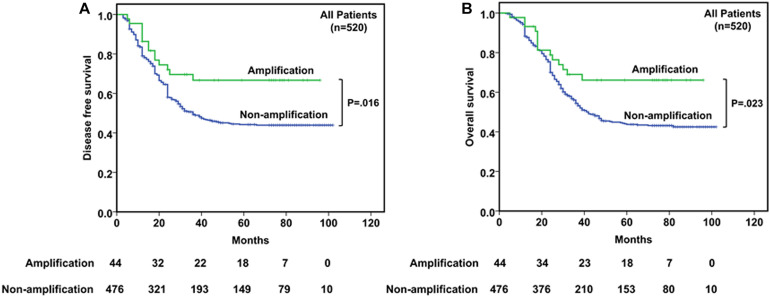
Kaplan–Meier curves of disease-free survival (DFS) **(A)** and overall survival (OS) **(B)** according to *CDK4* amplification status in 520 esophageal squamous cell cancer (ESCC) patients.

The 5-year DFS and OS rates for all patients were 32.1% and 32.9%, respectively, with a median follow-up period of 35.5 months (range, 3–102 months). Mean and median times to DFS were 41.6 and 31.0 months, while to OS were 44.9 and 35.5 months, respectively. Instances of disease progression (278), including 106 local recurrences and 172 lymph node or distant metastasis, were documented, and 277 patients (53.3%) died during the follow-up, in which 269 patients (51.7%) died of EC. To further explore the prognostic significance of *CDK4* amplification and clinical outcomes, Kaplan–Meier analysis with log-rank test was used to compare differences between subgroups. The Kaplan–Meier curves revealed that the *CDK4* amplification group with a median DFS and OS of 42.5 and 46.0 months, respectively, gained significant survival benefit compared with the group without *CDK4* amplification (median DFS, 30.0 months, *P* = 0.016; median OS, 35.0 months, *P* = 0.023) (Figure 2). Univariate analysis of prognostic significance revealed that grade, invasive depth, vessel invasion, nerve invasion, lymph node metastasis, clinical stage, and *CDK4* amplification were significantly associated with DFS and OS. In the multivariate analysis, invasive depth (*P* = 0.006, HR: 1.560, 95% CI: 1.133–2.149 for DFS; *P* = 0.008, HR: 1.542, 95% CI: 1.119–2.125 for OS) and *CDK4* amplification (*P* = 0.015, HR: 0.512, 95% CI: 0.299–0.877 for DFS; *P* = 0.021, HR: 0.530, 95% CI: 0.309–0.908 for OS) were associated with DFS and OS (Table 2).

**TABLE 2:** Univariate and multivariate survival analyses for DFS and OS in full cohort of patients with ESCC.

Variable	DFS		OS	
		
	*P*-value	Hazard ratio	*P-*value	Hazard ratio
		(CI 95%)		(CI 95%)
**Univariate analysis**				
Sex	0.109	1.299 (0.943–1.790)	0.128	1.283 (0.931–1.767)
Age	0.848	0.977 (0.768–1.243)	0.888	1.017 (0.800–1.295)
Grade	**0.032**	1.269 (1.021–1.577)	**0.045**	1.250 (1.005–1.554)
Invasive depth	**< 0.001**	1.921 (1.446–2.553)	**< 0.001**	1.952 (1.469–2.593)
Vessel invasion	**0.002**	1.536 (1.175–2.007)	**0.001**	1.562 (1.195–2.042)
Nerve invasion	**0.008**	1.394 (1.091–1.782)	**0.002**	1.460 (1.142–1.865)
Lymph node metastasis	**< 0.001**	2.809 (2.192–3.600)	**< 0.001**	2.854 (2.227–3.658)
Clinical stage	**< 0.001**	2.844 (2.222–3.639)	**< 0.001**	2.882 (2.252–3.687)
Site	0.980	0.997 (0.810–1.228)	0.813	1.026 (0.832–1.265)
Smoking	0.199	1.173 (0.919–1.495)	0.192	1.176 (0.922–1.499)
*CDK4* amplification	**0.020**	0.529 (0.309–0.906)	**0.027**	0.546 (0.319–0.934)
**Multivariate analysis**				
Grade	0.438	1.093 (0.873–1.367)	0.594	1.063 (0.849–1.331)
Invasive depth	**0.006**	1.560 (1.133–2.149)	**0.008**	1.542 (1.119–2.125)
Vessel invasion	0.977	0.996 (0.749–1.324)	0.907	1.017 (0.766–1.351)
Nerve invasion	0.964	1.006 (0.771–1.313)	0.677	1.058 (0.810–1.382)
Lymph node metastasis	0.192	1.980 (0.709–5.524)	0.158	2.095 (0.750–5.849)
Clinical stage	0.583	1.333 (0.478–3.721)	0.656	1.263 (0.452–3.528)
*CDK4* amplification	**0.015**	0.512 (0.299–0.877)	**0.021**	0.530 (0.309–0.908)

*ESCC, esophageal squamous cell carcinoma; CI, confidence interval; DFS, disease-free survival; OS, overall survival.*

*The features/variables that make significant contribution are in bold.*

### Survival Analyses Based on Clinical Stage

**FIGURE 3:**
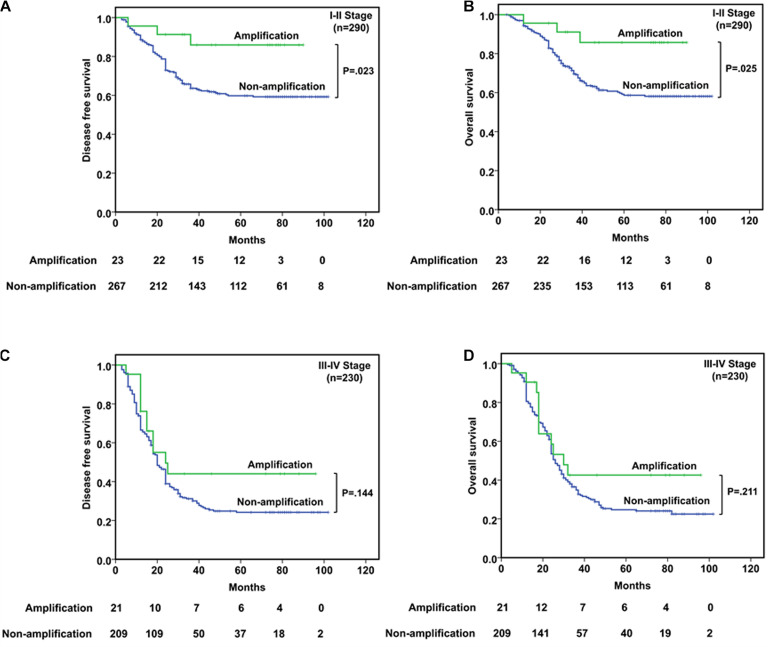
Survival analyses based on clinical stage of ESCC patients. **(A,B)** In stages I–II patients, *CDK4* amplification was significantly associated with better DFS (*P* = 0.023) and OS (*P* = 0.025). **(C,D)** In stages III–IV patients, *CDK4* amplification could not predict the prognosis in DFS (*P* = 0.144) or OS (*P* = 0.211).

In stages I–II patients (*n* = 290, Figures 3A,B), *CDK4* amplification was significantly associated with better DFS (*P* = 0.023) and OS (*P* = 0.025). Among the 23 patients with *CDK4* amplification, a better prognosis was observed, with a median DFS and OS being both 73.0 months compared with 45.0 and 47.0 months for 267 patients without *CDK4* amplification. As to the stages III–IV patients (*n* = 230, Figures 3C,D), *CDK4* amplification did not play the prognostic role whether in DFS (*P* = 0.144) or in OS (*P* = 0.211), since the median DFS and OS were 18.0 and 25.0 months, respectively, in 21 patients with *CDK4* amplification, whereas it was 20.0 and 25.0 months, respectively, for 209 patients without *CDK4* amplification.

## Discussion

Prognosis prediction and treatment guidance for ESCC are currently based on the TNM staging system, which provides prognostic information, and it will continue to be the most commonly applied approach for a fairly long time (Rustgi and El-Serag, 2014). However, patients with the same TNM stage may display different molecular phenotypes and prognoses. Many non-anatomic prognostic factors, especially genetic and molecular markers critical in carcinogenesis and cancer progression, are also found to have great significance in patient prognosis (Cao et al., 2014; Lin et al., 2017; Mei et al., 2017; Wang et al., 2017; Bi et al., 2020). Therefore, it is of great importance to identify accurate biological markers for the prognosis of ESCC, which may help subdivide patients at the same stage into different groups according to their prognosis. A better understanding of patient prognosis would help guide more personalized treatment for ESCC patients after curative surgery.

Aberrant *CDK4* amplification in malignant tissues has been reported to be involved in the development and progression of various cancers including liposarcoma (Creytens et al., 2015), glioblastomas (Schmidt et al., 1994), breast cancer (Piezzo et al., 2020), ovarian cancer (Masciullo et al., 1997), and melanoma (Muthusamy et al., 2006) through the cyclin D1–CDK4/6–Rb pathway. Ricciotti et al. (2017) performed a cut point analysis of the prognostic significance of *CDK4* amplification in patients with dedifferentiated liposarcoma by comparison of Kaplan–Meier survival curves using log rank tests. The study showed that *CDK4* amplification was associated with decreased DFS (*P* = 0.0169) and disease-specific survival (DSS) (*P* = 0.0140). Saada-Bouzid et al. (2015) also demonstrated that *CDK4* amplification was significantly associated with shorter recurrence-free survival, and overall survival in dedifferentiated liposarcoma patients. Altogether, the amplification of *CDK4* appears to be a negative event in liposarcoma. In glioblastoma patients, Fischer et al. (2010) reported that lack of amplification of *CDK4* was recognized to be associated with a significant longer survival time.

In the present study, we investigated *CDK4* amplification and its value in the prediction of survival in patients with ESCC. The correlation between *CDK4* amplification and the clinicopathological parameters of ESCC patients was also analyzed. Different from a singular criterion only using *CDK4/CEP12* ratio or *CDK4* copy numbers, we applied a more sophisticated *CDK4* FISH criterion considering percentage of *CDK4* clusters at the same time. Patients with *CDK4* amplification and non-amplification account for 8.5% (*n* = 44) and 91.5% (*n* = 476) of all the 520 ESCC patients, respectively. *CDK4* amplification rate (8.5%) determined by FISH analysis in our study is comparative with that of a previous study obtained by high-throughput sequencing methods (Song et al., 2014). There was no significant difference between *CDK4* amplification and *CDK4* non-amplification group regarding sex, age, grade, invasive depth, vessel and nerve invasions, lymph node metastasis, anatomic site, smoking, and clinical stage, which is in line with the conclusion that no significant associations were found between *CDK4* gene amplification and patient’s age, tumor size, and lymph node status in breast cancer (An et al., 1999).

Although there was no statistical significance, *CDK4* gene amplification was less common in tumors with higher histological grade. Moreover, it is worth noting that *CDK4* amplification had a significant negative correlation with disease progression (*P* = 0.003) and death (*P* = 0.006) (Table 1). *CDK4* seems to be negatively correlated with some indicators indicating poor prognosis in ESCC. Interestingly, different from the prognosis value of *CDK4* amplification in dedifferentiated liposarcoma and glioblastoma patients, we demonstrated that *CDK4* amplification was associated with a better DFS (*P* = 0.016) and OS (*P* = 0.023) (Figure 2). Furthermore, *CDK4* amplification was not a common genetic alteration but proved to be an independent prognostic marker in patients with ESCC (Table 2). The results of this study seem to be opposite to the prognosis of other tumor types. This may be as a result of the complexity of the gene regulation process in ESCC. The occurrence and development of ESCC is a multistage and multifactor process, which involves the interaction of multiple oncogenes and tumor suppressor genes (Gao et al., 2014; Lin et al., 2014; Cancer Genome Atlas Research Network, Analysis Working Group: Asan University, Bc Cancer Agency, Brigham and Women’s Hospital, Broad Institute, Brown University, et al., 2017). In addition, we speculate that it may be due to the influence of cancer species, and geographical and environmental factors; the causes of different tumors are not the same, leading to the differences in research results. To the best of our knowledge, this study is the first to evaluate the value of *CDK4* amplification as a novel candidate prognostic biomarker in patients with ESCC, so it is necessary to further investigate the upstream and downstream genes of *CDK4* to clarify its role and elucidate the prognostic utility in ESCC.

Given that clinical stage is an important clinicopathological factor, the prognosis usually varies between patients with different stages. Therefore, we categorized the patients into the I–II stage group and III–IV stage group. In the I–II stage group, *CDK4* amplification was significantly associated with both better DFS and OS compared with the non-amplification group. However, this significant correlation was not found in the III–IV stage patients implying that prognostic value of *CDK4* amplification is relying on clinical stage (Figure 3). It is suggested that *CDK4* may change in the early stage of ESCC and play an important role in the occurrence and development of the disease. With the increase in clinical stage, more and more genes in ESCC are changed (Sudo et al., 2019), and the interaction between genes becomes complex, which affects the role of *CDK4*.

In summary, we have first proved the prognostic significance of *CDK4* amplification as a favorably independent prognostic factor for DFS and OS in Chinese patients with ESCC. Combining *CDK4* amplification with the TNM staging system might add more information to better predict the prognosis of ESCC patients.

## Data Availability Statement

The original contributions presented in the study are included in the article/supplementary material, further inquiries can be directed to the corresponding author/s.

## Ethics Statement

The studies involving human participants were reviewed and approved by the Human Research Ethics Committee of Zhongshan Hospital, Fudan University. The patients/participants provided their written informed consent to participate in this study.

## Author Contributions

JH wrote the draft of the manuscript. XW and YH evaluated CDK4 copy numbers. XZ, WC, and LL analyzed the results of the experiment. QS, HW, and JL constructed Tissue microarrays (TMA). LX, YX, and LS were involved in the picture editing. LT and DJ put forward suggestions for improvement. YH and JS revised the manuscript. All authors contributed to the article and approved the submitted version.

## Conflict of Interest

The authors declare that the research was conducted in the absence of any commercial or financial relationships that could be construed as a potential conflict of interest.
